# ‘You Learn How to Hate’: Adapting a Healthy Relationship Curriculum Using a Trauma-Informed Race Equity Lens

**DOI:** 10.3390/ijerph18189916

**Published:** 2021-09-21

**Authors:** Shannon Guillot-Wright, Elizabeth D. Torres, Bianca Obinyan, Jeff R. Temple

**Affiliations:** 1 Center for Violence Prevention, University of Texas Medical Branch, Galveston, TX 77555-0587, USA; edtorres@utmb.edu (E.D.T.); jetemple@utmb.edu (J.R.T.); 2 School of Medicine, University of Texas Medical Branch, Galveston, TX 77555-0587, USA; boobinya@utmb.edu

**Keywords:** structural violence, interpersonal violence, race equity, trauma informed, healthy relationships, school curriculum

## Abstract

Teen dating violence is a public health concern that can lead to short- and long-term mental and physical health consequences, including depression, anxiety, risky behaviors, and unhealthy future relationships. Research shows that social and structural determinants of health, such as racism, low socio-economic status, and neighborhood conditions, may predispose certain communities to violence. To better understand methods to reduce TDV among ethnically and economically diverse populations, we used a trauma-informed race equity lens to adapt an efficacious prevention program known as *Fourth R*. This universal program has been shown to reduce some dating violence, substance use, and risky sexual behaviors, but there remains room for improvement. Specifically, more attention to trauma and the importance of societal risk and protective factors may improve the program’s effectiveness. Thus, focus group discussions were conducted with students and we then adapted *Fourth R* lessons specific to trauma, racism, and discrimination. Major themes discussed are that *Fourth R* and other prevention programs should focus attention on social and structural issues, such as racism and discrimination.

## 1. Introduction

Teen dating violence (TDV) is an increasingly recognized public health concern linked to short- and long-term mental and behavioral health consequences, such as depression, anxiety, risky sexual behavior, substance use, and unhealthy relationships [1,2,3]. In the US, over twenty states have laws requiring school districts to incorporate TDV prevention programming; however, these mandates typically come with little or no funding and little guidance on implementation [4]. Moreover, existing TDV prevention programs generally lack lessons that incorporate structural and social factors, such as childhood trauma, racism, and discrimination [5]. The aim of this brief report is to present pilot data related to the augmentation process of a healthy relationship program based on student perceptions of trauma, racism, and discrimination.

### Study Overview

Research collected over the course of nearly three decades has found that children and youths who experience adverse childhood experiences (ACEs) are at heightened risk for short- and long-term physical and mental health consequences [6]. ACEs traditionally include childhood abuse, neglect, familial incarceration/separation, and food insecurity [6]. More recent attention has focused on the impact of adverse childhood and community experiences (ACCEs), which includes inequitable access to high-quality health care, education, employment, and housing, specifically for Black, Indigenous, People of Color (BIPOC) [7]. Indeed, others have argued that, while these outer-levels of the socio-ecological framework are as important as the inner layers (e.g., behavior change; knowledge and skills), they are largely ignored in prevention and intervention. [8,9].

As a complement to focusing on the outer layers of social influence, it is also important to understand dating violence and trauma through a critical race theory (CRT) lens. CRT notes that race is a socially constructed notion and that it is the embodiment of racism, not race, that impacts the mental and physical health outcomes of people and groups [10]. Taken together, studying TDV from a trauma-informed race equity lens within the educational system, while also taking structural and systemic factors into account, is an innovative and necessary approach to preventing violence. Our approach addresses the ways that racism and discrimination are normalized in relationships and the educational system, as well as how students of color negotiate and defy stereotypes.

The healthy relationship program we augmented from a trauma informed race equity lens was *Fourth R*, which references the missing R, relationships, that can be taught in the same way reading, writing, and arithmetic are taught in a school-based system [11]. The program teaches healthy relationship skills through a universal approach and targets shared risk and protective factors of multiple problem behaviors (for an in-depth evaluation of *Fourth R*, see Wolfe et al., 2009) [11]. While *Fourth R* has been shown to effectively reduce risky and promote healthy behaviors, there is room for improvement; namely, increasing attention to childhood trauma and emphasizing the importance of community and societal risk and protective factors [5].

## 2. Methods

### 2.1. Participants and Sampling

The pilot project was conducted using focus group discussions (FGDs) with students receiving *Fourth R* through a community-based participatory research (CBPR) partnership. The FGDs included ten high school students of diverse grade levels, ethnicities, and backgrounds. To maximize comfort and privacy, CBPR FGDs occurred at a time (e.g., during school hours) and a location (e.g., empty classroom, school library) that were both convenient for participants without school teachers or administrators present. Student recruitment for CBPR happened through pre-existing community collaboration and approval from school administrators and teachers. Initially, a trusted teacher and collaborator on the project asked for volunteers from classes, and then snowball sampling was used. Students who volunteered understood their grades would not be impacted by participation. Following CBPR best practices, collaborators continued to meet with students for one hour each week for two consecutive school years to inform how the healthy relationships curriculum could be best implemented in schools [12]. Weekly discussions were not recorded, but field notes were taken.

### 2.2. Procedure

CBPR is an increasingly used approach to encourage populations to share, discuss, and analyze their problems and identify solutions. Using a combination of research and advocacy, the goal of CBPR is to collect community data while simultaneously strengthening community voices [12]. As CBPR is a collaborative approach that involves all participants in the research process, we ensured that student voices were centered in the augmentation of *Fourth R*. The FGDs were conducted by community collaborators. A secondary data analysis of the FGDs was approved by the University of Texas Medical Branch’s Institutional Review Board following ethical principles outlined in the Belmont Report.

### 2.3. Measure

In-depth interview guides to facilitate rapport and trust with the participants were developed [13]. A standardized protocol was followed with the following components: (a) explanation of study, participant’s right to stop the interview at any time, and the study’s confidential nature; (b) use of recorders; (c) reason for taking notes; and (d) importance of not using any specific names of anyone involved in any illegal activities. Further, a trusting and respectful environment was created to maximize the likelihood that confidentiality was assured, and that participants felt comfortable talking freely and openly during the FGDs. Following the FGDs, the interviewer documented any additional notes from the session [13]. FGD questions focused on past experiences with healthy relationship programming, what relationships mean to them, how they obtain information on healthy relationships, relationship topics they want to learn more about, and ended with students giving ideas for what to include/exclude if they were in charge of designing a healthy relationship program. Prior to adapting *Fourth R* lessons, identified schools and teachers were actively facilitating the original *Fourth R* for at least one full academic year.

### 2.4. Data Analysis

Qualitative analyses were guided by an inductive approach to identify key themes and discourses that emerged from narrative data, followed by an interpretive analysis that explored participants’ language and social interactions [14]. First, transcripts were read and reread several times by the research team to become familiar with the data. Next, a thematic coding of the FGDs was undertaken. Specifically, we used an open coding strategy to identify themes regarding interactions, attitudes, and experiences with healthy relationship curriculum and programming. Then, the interpretation of participant’s narratives was informed by existing typologies, and thematic codes inductively emerged from the data [15]. An inductive and interpretive approach allowed us to explore social interactions and better understand the relationship between actors. All data were analyzed in Atlas.ti Cloud.

## 3. Results

Themes that emerged from the student CBPR FGDs were that healthy relationship programming should: (1) incorporate social media (e.g., Instagram), contemporary posters (e.g., bright colors; diverse photographs of adolescents), and theater/film (e.g., school play or YouTube videos) instead of large school assemblies; (2) begin in middle school; (3) focus on cyberbullying (e.g., racist comments through social media); and (4), important to this paper, focus attention on social and structural issues, such as racism and discrimination in school generally and within relationships specifically.

Students provided detailed accounts of their experiences with racism and how it related to bullying, suicidal ideation, low self-esteem, depression, and social anxiety. One student in the FGD replied, ’Sometimes you feel like you’re not good enough because [of] your color. Sometimes you hate yourself, not hate yourself, you just don’t think you’re enough’. Other students discussed their experience being called racist, homophobic, and xenophobic slurs, such as a student who said, ‘I’m mixed, so I’m called cracker and gringo … and also called illegal immigrant’. Over half of the students in the focus group gave detailed accounts of being called racist slurs in school or bullied for not talking a certain way. Other students who did not talk about past experiences raised their hands when asked, ’Who else [experienced racism and discrimination]?’ Although racism was the major factor discussed, students also mentioned discrimination based on their sexuality. Based on these responses, the lead researcher asked students how they would improve these issues in their school, and the consensus was that school administrators and teachers needed to directly address racism and discrimination. As one student noted, ‘You learn how to hate’.

## 4. *Fourth R* Adaptation Based on Results

Following a CBPR approach that views all participants as equal partners [16,17], we incorporated the students’ suggestions to adapt *Fourth R* by including activities that addressed trauma, racism, gender discrimination, and other social and structural factors that may lead to unhealthy relationships. Adaptations were added throughout the curriculum using a culturally responsive teaching approach, which focuses on how to educate a diverse student body and create an inclusive curriculum [18]. In accordance with best practice, we conducted an in-depth analysis and revision of instructional materials, including visual aids, students’ handouts, teacher reference sheets, and lesson plan instructions (see Table 1). For example, we augmented the intervention to address sexuality, racism, micro-assaults (e.g., using derogatory terms for ethnic minorities), micro-insults (e.g., questioning how a person of color obtained a particular job or place at a particular university), and micro-invalidations (e.g., denying others’ experiences of racism or accusing them of being oversensitive) [19].

Furthermore, the curriculum adaptation is inclusive of trauma-informed activities that address previously experienced trauma. Research has long shown that there is a strong association between a child who experiences multiple adverse childhood experiences (e.g., neglect, violence, economic insecurity) and subsequent sexual risk taking, poor mental health, problematic substance use, and interpersonal and self-directed violence [20,21]. Our curriculum adaptations provide teachers the opportunity to understand students’ home environments, while also validating students’ emotions and life experiences. By addressing and acknowledging the outer layers of the social ecology, we believe lessons targeting individual behavior change will be more effective.

## 5. Discussion

While students were receptive to participating in *Fourth R*, the CBPR discussions revealed that students preferred a holistic approach that included all layers of the social-ecological model. Students understood relationship health to include all relationships and emphasized the importance of considering social and structural factors, such as past trauma, racism, and discrimination in preventing violence, abuse, and unhealthy relationships. For solutions, students suggested including explicit discussions about racism and discrimination in school and through the relationship curriculum.

It is also important to note that embedding equity into school mental health programming in a responsible and conscientious manner requires an influx of resources—time and funding for training, support, and educational development. Time and funding, in turn, depend on support from school and public leaders, including school superintendents and local, state, and federal policymakers. Without support from leadership and continued education, students who have already experienced racism and discrimination stand to experience other forms of harm or inequity because of a lack of training or continued follow-up. While ambitious, these steps are necessary to prevent further injustice, attend to previous injustice, and move forward towards a more equitable society. Indeed, these efforts take long-term commitment, community collaboration, and patience.

Efforts to make schools and school mental health more equitable will also result in setbacks and mistakes. Improvements in integrating a trauma-informed race equity framework will vary between districts and even schools, but the lessons learned in this study can provide some insights to other districts wanting to expand their health equity efforts. Lessons include (1) involving students in the discussions and implementation of school-based programming, taking their values and lived experiences seriously and into account; (2) understanding the intersectionality of race/ethnicity, gender, sexuality, and socioeconomic status and how they impact learning outcomes; and (3) attending to the long-term impact of social, political, and economic factors on mental and physical health through a school-based curriculum.

## 6. Limitations

We accomplished our main goal of augmenting and implementing a race equity, trauma-informed program. The additions were well received by stakeholders and provided an avenue to address gaps in the curriculum. However, due to COVID-19-related school closures, we were unable to preliminarily evaluate the adapted intervention. One teacher piloted units 1, 2, and 4 of the augmented *Fourth R*, reaching approximately 123 students over five class periods; however, the COVID-19 pandemic interrupted the implementation of unit 3 and evaluation of the piloted program.

## 7. Conclusions

Teen dating violence is an ongoing public health concern that negatively impacts youths’ mental and physical wellbeing. The evidence-based *Fourth R* curriculum demonstrates encouraging results in addressing TDV through positive youth development and targeting shared risk and protective factors of multiple problem behaviors. However, students in CBPR discussions highlighted the lack of attention paid to social and structural factors within the curriculum, such as racism and discrimination. Therefore, we augmented the curriculum to include lessons that addressed trauma, racism, and other structural and social factors. The adapted curriculum provides an opportunity for students to reflect on how race/ethnicity, gender, sexuality, and socioeconomic status play a role in their relationships with peers and romantic partners. The augmentation also offers support for teachers to address these issues while providing tools for them to validate students’ circumstances and provide referrals. Future research should test whether the augmented *Fourth R* intervention effectively reduces violence, over and above the standard curriculum.

## Figures and Tables

**Table 1:** Augmented *Fourth R* Lessons.

	Example Lesson	Curriculum	Adaptation
Unit 1: Personal Safety and Injury Prevention	Rule of Fives	Students recognize what matters in the present moment may not matter as much in five days, five weeks, five months, or five years (e.g., breaking up with a boyfriend; not receiving an A on a test).	Students encouraged to think of a circumstance where the Rule of Fives may not apply and have life-long consequences (e.g., witnessing severe violence; experiencing racism)
Unit 2: Substance Use, Addictions, and Related Behaviors	Stand on the Line and Communication Line activities	Students discuss influences that predispose or deter them from substance use and addictive behaviors (e.g., peer pressure).	The two activities are replaced with; Take One Step Forward’, which allows students to actively engage and reflect on how substance use can be related to individual, social, and structural factors (e.g., a student is left unattended after school because her parent works multiple jobs).
Unit 3: Human Development and Sexual Health	Stereotypes and support	Students discuss skills and strategies to build a healthy relationship as well as factors that influence our understanding of gender identity, sexual orientation, and stereotypes.	Students learn about societal assumptions when defining masculinity, femininity, and heterosexual relationships and discuss comfort level with various social situations (e.g., ‘you see a little boy playing with a princess barbie’; ‘your Social Studies teacher is a Black person’).
Unit 4: Healthy Eating	How does culture/family background impact food choices?	Students answer questions on how parent/guardian work schedule, family finance, geographical location, religious and cultural background and peers influence food choices.	An additional point is added to be sensitive to other people’s food choices when suggesting healthier alternatives (e.g., think about what the person currently eats and modify it, such as eating smaller portions, instead of suggesting they eat what you eat).

## Data Availability

All requests for data should be sent electronically to the corresponding author.

## References

[1] Exner-Cortens D., Eckenrode J., Rothman E. (2012). Longitudinal Associations Between Teen Dating Violence Victimization and Adverse Health Outcomes. Pediatrics.

[2] Temple J.R., Freeman D.H. (2011). Dating violence and substance use among ethnically diverse adolescents. J. Interpers. Violence.

[3] Vives-Cases C., Davo-Blanes M.C., Ferrer-Cascales R., Sanz-Barbero B., Albaladejo-Blázquez N., Segundo M.S.-S., Lillo-Crespo M., Bowes N., Neves A.S., Mocanu V. (2019). Lights4Violence: A quasi-experimental educational intervention in six European countries to promote positive relationships among adolescents. BMC Public Health.

[4] Cornelius T.L., Resseguie N. (2007). Primary and secondary prevention programs for dating violence: A review of the literature. Aggress. Violent Behav..

[5] Guillot-Wright S., Lu Y., Torres E.D., Macdonald A., Temple J.R., Geffner R., Vieth V., Vaughan-Eden V., Rosenbaum A., Hamberger L., White J. (2020). Teen Dating Violence Policy: An Analysis of Teen Dating Violence Prevention Policy and Programming. Handbook of Interpersonal Violence Across the Lifespan.

[6] Felitti V.J., Anda R.F., Nordenberg D., Williamson D.F., Spitz A.M., Edwards V., Koss M.P., Marks J.S. (1998). Relationship of Childhood Abuse and Household Dysfunction to Many of the Leading Causes of Death in Adults: The Adverse Childhood Experiences (ACE) Study. Am. J. Prev. Med..

[7] Ellis W.R., Dietz W.H. (2017). A New Framework for Addressing Adverse Childhood and Community Experiences: The Building Community Resilience Model. Acad. Pediatr..

[8] Lounsbury D.W., Mitchell S.G. (2009). Introduction to Special Issue on Social Ecological Approaches to Community Health Research and Action. Am. J. Community Psychol..

[9] Wendel M.L., Nation M., Williams M., Jackson T., Jones G., Debreaux M., Ford N. (2020). The structural violence of white supremacy: Addressing root causes to prevent youth violence. Arch. Psychiatr. Nurs..

[10] Dutil S. (2020). Dismantling the School-to-Prison Pipeline: A Trauma-Informed, Critical Race Perspective on School Discipline. Child. Sch..

[11] Wolfe D.A., Crooks C., Jaffe P., Chiodo D., Hughes R., Ellis W., Stitt L., Donner A. (2009). A school-based program to prevent adolescent dating violence: A cluster randomized trial. Arch. Pediatrics Adolesc. Med..

[12] Faridi Z., Grunbaum J.A., Gray B.S., Franks A., Simoes E. (2007). Community-based Participatory Research: Necessary Next Steps. Prev. Chronic Dis..

[13] Krueger R.A., Casey M.A. (2009). Developing a Questioning Route. Focus Groups: A Practical Guide for Applied Research.

[14] Van Dijk T.A. (2011). Discourse Studies: A Multidisciplinary Introduction.

[15] Davies C.A. (2008). Reflexive Ethnography: A Guide to Researching Selves and Others.

[16] Gower A.L., Valdez C.A.B., Watson R.J., Eisenberg M.E., Mehus C.J., Saewyc E.M., Corliss H., Sullivan R., Porta C.M. (2019). First- and Second-Hand Experiences of Enacted Stigma Among LGBTQ Youth. J. Sch. Nurs..

[17] Madubata I., Spivey L.A., Alvarez G.M., Neblett E.W., Prinstein M.J. (2021). Forms of Racial/Ethnic Discrimination and Suicidal Ideation: A Prospective Examination of African-American and Latinx Youth. J. Clin. Child Adolesc. Psychol.

[18] Gay G. (2002). Preparing for Culturally Responsive Teaching. J. Teach. Educ..

[19] West K. (2019). Testing Hypersensitive Responses: Ethnic Minorities Are Not More Sensitive to Microaggressions, They Just Experience Them More Frequently. Pers. Soc. Psychol. Bull..

[20] Hughes K., A Bellis M., A Hardcastle K., Sethi D., Butchart A., Mikton C., Jones L., Dunne M.P. (2017). The effect of multiple adverse childhood experiences on health: A systematic review and meta-analysis. Lancet Public Health.

[21] Shorey R.C., Fite P.J., Choi H.J., Cohen J.R., Stuart G.L., Temple J.R. (2015). Dating Violence and Substance Use as Longitudinal Predictors of Adolescents’ Risky Sexual Behavior. Prev. Sci..

